# Enhanced prediction of stock markets using a novel deep learning model PLSTM-TAL in urbanized smart cities

**DOI:** 10.1016/j.heliyon.2024.e27747

**Published:** 2024-03-13

**Authors:** Saima Latif, Nadeem Javaid, Faheem Aslam, Abdulaziz Aldegheishem, Nabil Alrajeh, Safdar Hussain Bouk

**Affiliations:** a Department of Management Sciences, COMSATS University Islamabad, Islamabad 44000, Pakistan; b Department of Computer Science, COMSATS University Islamabad, Islamabad 44000, Pakistan; c International Graduate School of Artificial Intelligence, National Yunlin University of Science and Technology, Yunlin, 64002, Taiwan; d School of Business Administration (SBA), Al Akhawayn University, Ifrane, 53003, Morocco; e Department of Urban Planning, College of Architecture and Planning, King Saud University, Riyadh, 11574, Saudi Arabia; f Department of Biomedical Technology, College of Applied Medical Sciences, King Saud University, Riyadh, 11633, Saudi Arabia; g Old Dominion University, Hampton Blvd, Norfolk, 4114, United States

**Keywords:** Bayesian optimization, Contractive autoencoder, Ensemble empirical mode decomposition, Peephole LSTM, Stock market prediction, Temporal attention layer, Time series, Urban planing

## Abstract

Accurate predictions of stock markets are important for investors and other stakeholders of the equity markets to formulate profitable investment strategies. The improved accuracy of a prediction model even with a slight margin can translate into considerable monetary returns. However, the stock markets' prediction is regarded as an intricate research problem for the noise, complexity and volatility of the stocks' data. In recent years, the deep learning models have been successful in providing robust forecasts for sequential data. We propose a novel deep learning-based hybrid classification model by combining peephole LSTM with temporal attention layer (TAL) to accurately predict the direction of stock markets. The daily data of four world indices including those of U.S., U.K., China and India, from 2005 to 2022, are examined. We present a comprehensive evaluation with preliminary data analysis, feature extraction and hyperparameters' optimization for the problem of stock market prediction. TAL is introduced post peephole LSTM to select the relevant information with respect to time and enhance the performance of the proposed model. The prediction performance of the proposed model is compared with that of the benchmark models CNN, LSTM, SVM and RF using evaluation metrics of accuracy, precision, recall, F1-score, AUC-ROC, PR-AUC and MCC. The experimental results show the superior performance of our proposed model achieving better scores than the benchmark models for most evaluation metrics and for all datasets. The accuracy of the proposed model is 96% and 88% for U.K. and Chinese stock markets respectively and it is 85% for both U.S. and Indian markets. Hence, the stock markets of U.K. and China are found to be more predictable than those of U.S. and India. Significant findings of our work include that the attention layer enables peephole LSTM to better identify the long-term dependencies and temporal patterns in the stock markets' data. Profitable and timely trading strategies can be formulated based on our proposed prediction model.

## 1 Introduction

Stock markets play an inevitable functional role for the growth of an economy by efficient allocation of resources and creation of liquidity in the urbanized smart cities. They are the formal mechanisms for fulfilling the capital needs of individuals, companies and institutions. At present, around sixty stock exchanges worldwide represent approximately $125 trillion of the total market capitalization of global equity markets. The U.S. stock market is the world's largest equity market having a total share of 42% followed by China with a share of around 8% [1]. The empirical analysis of equity markets is an important research area as billions of dollars are being traded on daily basis in these markets.

In literature, technical and fundamental analyses are the two major methods for investigating stock markets. Technical analysis is performed using historical data of stocks' prices and technical indicators. It is more useful for short-term predictions and trading strategies. While, fundamental analysis is based on information about companies and economy, and is more oriented towards long-term forecasts and investment strategies. Technical analysis can be combined with fundamental analysis for long-term predictions [2]. The stock market forecast can be made using classical statistical methods like regression, Autoregressive Moving Average (ARMA), Autoregressive Integrated Moving Average (ARIMA) and Generalized Autoregressive Conditional Heteroskedasticity (GARCH), and machine learning (ML) methods like Support Vector Machine (SVM), Decision Tree (DT) and Random Forest (RF) [4], [5], [3]. These methods have been extensively used for predicting financial time series, however, they have some limitations which make them inappropriate for sequential time series data. For instance, the classical statistical methods are linear models, assume data to be non-stationary and predict target variable based on recent samples only [6]. ML algorithms address these problems as they are non-linear and do not assume particular data distributions. However, many ML models have problem of short-term dependency [7]. Over recent decades, the Recurrent Neural Network (RNN) based models have been extensively used to generate valid forecasts. They too are prone to the problems of overfitting and vanishing gradients [8]. Thus, there remains the debate that which methods or hybrid models can provide reliable and efficient prediction of stock markets. Researchers in the field of finance have been looking for better prediction models to narrow this research gap. In this regard, this study uses the RNN-based model Long Short Term Memory (LSTM) for studying predictability of prices in stock markets.

The empirical analysis of stock markets is not a trivial task. A stock market is affected by various diversified factors including those of markets, industries, companies, economy, politics and globalization. These are not independent factors, rather they have very complex connections making the task of predicting the behavior of stock markets cumbersome [9] and [10]. The long-lasting debate over the effective and accurate prediction methods of stock prices has never been concluded because financial time series are noisy and non-stationary and therefore it is difficult to predict the future behavior of stock prices [11]. However, a correct prediction of stocks' future movements is sacred for investors as they can translate it into huge profits. Investors are interested in reliable forecasts of equity markets for formulating trading strategies with higher capital gains and lower risks [12] and [13]. Therefore, there remains a strong motivation in research and academia for constantly working upon the development of more advanced forecasting algorithms for profitable investment strategies [14]. Few successful developments beating the existing algorithms can change the landscape of the algorithmic trading in equity markets. Moreover, the robust prediction models can facilitate the creation of certain peepholes for the stakeholders of stock markets to visualize the probable outcomes and plan accordingly. For instance, the authors in a recent study [15] focused on the problems of noisy data and poor generalization performance of the stock prediction models using historical trading data. The Deep Learning (DL) methods of autoencoder (AE) and Mixture Density Network (MDN) were separately used to obtain trading mode deviation and price prediction uncertainty of the samples. Thereafter, the curriculum learning algorithm was applied to train the model which led to the improved micro-F1 metric and also alleviated the class imbalance issue.

The main objective of our research work is twofold. First is to explore the utility of various ML methods for analyzing the time series data of stock market. In this regard, literature has been accessed for contemporary algorithms of dimensionality reduction, time series decomposition and hyperparameters' optimization. Accordingly, a set of algorithms including ensemble empirical mode decomposition, contractive autoencoder and Bayesian optimization have been selected respectively for reducing noise of the price series, extracting important features and tuning of the hyperparameters of the proposed model. These methods are included to complement the prediction performance of the proposed DL model. Secondly, our objective is to capitalize on the potential of DL for accurate prediction of the future direction of stock markets. An accurate prediction model based on the analysis of technical indicators can better decide the entry and exit points of a stock market in the short run. This is crucial in algorithmic trading as the wrong timings of entering or exiting the market often leads to monetary losses for traders. Reliable stock market predictions help in articulating investment strategies to capitalize the potential of the market for gaining maximum profits that is the ultimate objective of stock trading.

A novel DL model Peephole Long Short-Term Memory (PLSTM) with a temporal attention layer is proposed to forecast the trend of stock markets. The peephole LSTM is chosen as the basic prediction model because of its ability to capture long-term dependencies pertinent to financial data. At first, the preprocessed time series data of prices is decomposed into orthogonal combination of their simpler components using the noise-assisted data analysis method of ensemble empirical mode decomposition. The component with the highest complexity, represented by sample entropy, is subtracted from the original price series to generate a filtered series with reduced complexity and noise [16]. Secondly, forty technical indicators are calculated from the stock prices data [17] and a contractive autoencoder algorithm is applied to extract the key features for improving prediction accuracy. Finally, the extracted features and the filtered price series are passed on to the peephole LSTM and then the TAL for accurate forecasts of market direction. TAL is introduced to select the relevant information with respect to time and enhance predictability of the proposed model. Meanwhile, the hyperparameters of the proposed deep model are optimized using Bayesian optimization. Moreover, the ML models SVM and RF and the DL models Convolutional Neural Network (CNN) and LSTM have been used as benchmark models for comparison with the proposed model. Experimental results prove the superior performance of the proposed model to the benchmark models. The prediction and forecasting terms are used interchangeably in this article. Moreover, Table 1 presents the list of acronyms.

**Table 1:** List of Acronyms.

Abbreviation	Full Form
AE	Autoencoder
AI	Artificial Intelligence
ANN	Artificial Neural Network
ARMA	Autoregressive Moving Average
ARIMA	Autoregressive Integrated Moving Average
AUC-ROC	Area Under Receiver Operating Characteristics Curve
CAE	Contractive Autoencoder
CNN	Convolutional Neural Network
DL	Deep Learning
EEMD	Ensemble Empirical Mode Decomposition
EMH	Efficient Market Hypothesis
FTSE	The Financial Times Stock Exchange
IMFs	Intrinsic Mode Functions
LSTM	Long-Short Term Memory
MCC	Matthew Correlation Coefficient
ML	Machine Learning
OHLCV	Open, High, Low, Close, Volume
PLSTM	Peephole Long Short Term Memory
PR-AUC	Area under Precision Recall Curve
RF	Random Forest
RNN	Recurrent Neural Network
RWH	Random Walk Hypothesis
SaEn	Sample Entropy
S&P	Standard and Poor's
SSE	Shanghai Stock exchange
SVM	Support Vector Machine
TAL	Temporal Attention Layer
TIs	Technical Indicators

### 1.1 Major contributions of the study

This study makes following contributions to the existing literature on predicting stock markets' trend.

• A novel DL based model Peephole LSTM with TAL (PLSTM-TAL) is proposed to predict the future direction of the sampled stock markets.
• A rich set of features including many technical indicators (TIs) calculated from the Open, High, Low, Close and Volume (OHLCV) is added in the feature space along with the features of Close price and Volume of the sampled stock indices in this study [17].
• The feature space is extracted using the contractive autoencoder algorithm [18] for significant attributes impacting the stock markets.
• The signal decomposition method of EEMD is applied to decompose the closing price series into its quasi-stationary components for mitigating the impact of noise and developing a robust prediction model. The reduced series is obtained after subtracting the component with highest sample entropy from the original series and is included in the feature space [16].
• Hyperparameters of the proposed PLSTM-TAL model are tuned using Bayesian optimization for parameters' tuning in an informed manner and for enhancing the prediction performance of the model [19] and [20].
• The proposed prediction model is compared with the ML techniques RF and SVM and the DL models CNN and LSTM with respect to their performance metrics.

## 2 Related work

The literature on financial markets has huge potential for developing new robust models to accurately predict trends of financial markets for facilitating their participants in timely and profitable decision making. The classical finance theories of Efficient Market Hypothesis (EMH) [21] and Random Walk Hypothesis (RWH) [22] posit that past prices (trends) cannot be used to predict future prices (trends) with more than 50% accuracy. Hence, both financial theories regard stock market as unpredictable system. However, many researchers and academicians have been analyzing the predictability of stock markets using various classical models of statistics and econometrics. Yet, these methods are not good at modeling the chaotic and non-linear structure of equity markets, plus they are slow in processing and handling large data with many features [23]. Moreover, the classical parametric models like ARMA and ARIMA require predefined parameters based on pre-assumptions about the data and consider only recent observations as inputs, thus they cannot well capture the long-term trends in the data [3] and [5].

An ideal prediction model is also capable of processing a large number of diverse features as inputs so that the intricate relationships among them can be modeled. Like in the study [24], a variety of time series, technical analysis and fuzzy logic models have been used to perform the challenging task of stock market forecasting. These models have been successful in their experimental settings, however these settings did not match the real-time dynamics of stock markets. Moreover, there are some drawbacks of the previously used models including (1) Strict and unrealistic statistical assumptions, (2) Biasness induced in forecasting due to human judgements, and (3) It is not easy to find a proper threshold, and the results are prone to experimental settings. Many ML and DL models can address these issues. Many ML techniques are well capable of modeling non-linear and non-stationary data, yet they have short-term dependencies and are not suitable for large data sets. Thereby, DL models can overcome the shortcomings of ML techniques and perform better while modeling large complex data. However, DL models are computationally more complex and require more time for processing the data. Hence, a trade-off between the prediction performance and computational complexity should be sought while selecting an appropriate prediction model [23].

The efficacy of the ML methods cannot be denied for analyzing the patterns of the less complex and low dimensional datasets. The stock market predictions are more accurate and accessible with ML techniques [25]. For instance, authors in [17] used tree-based ensemble models for predicting three stock exchanges (NYSE, NASDAQ, NSE) and found Extra Tree (ET) as the best classifier. In another research work [12], a decision support system was proposed for algorithmic trading by integrating Weighted Multicategory Generalized Eigenvalue SVM and RF algorithms (RF-WMGEPSVM) to generate buy-hold-sell signals. The authors preferred SVM over Artificial Neural Networks (ANNs) as SVM offers better generalization performance. Besides, there are studies contrasting the performance of both ML and DL models for their research problems. Likewise, [26] compared nine ML techniques – DT, RF, Adaptive Boosting (AdaBoost), Extreme Gradient Boosting (XGBoost), Support Vector Classifier (SVC), Naïve Bayes (NB), k-Nearest Neighbors (kNNs), Logistic Regression (LR) and ANNs with the DL models of RNNs and LSTM. Its results proved the superior performance of the deep models RNN and LSTM mover the ML techniques.

The trend of a stock market is affected by many diversified factors with complex and non-linear associations and traditional mathematical models find it difficult to determine this trend. Besides, fundamental and technical analyses are sensitive to the subjective judgement of stock markets' investors [27]. Therefore, the contemporary research on forecasting financial markets is applying these analyses in combination with ML [5] and DL [13] algorithms. Such studies provided empirical evidence of the superior performance of the ML and DL models. Moreover, the availability of high-frequency financial data over time encouraged the development of more efficient ML methods capable of processing big data. Thus, NNs became deeper with inclusion of more neurons intensive hidden layers to enhance their predictability for complex and large data. However, simple feed forward NNs could not handle sequential data. Thereby, RNNs were developed with memory structures to cater the processing needs of temporal data. Yet they had the issues of overfitting and short-term dependency due to their limited memory [8]. The development of gates mechanism in LSTM addressed these limitations. LSTMs are special RNNs having extended memory architectures suitable for processing longer sequences and predicting long-term trends. The structure of LSTM is capable of understanding the context-specific dependence of stock prices on their historical values which is crucial for accurate forecasts [28].

LSTM was used as a DL representative model in [13] to capture the long-range dependencies in the data. The results indicated that LSTM outperformed ARIMA with an 85% less RMSE. In another study [29], authors applied an LSTM DL framework for predicting the daily stock closing prices of Standard and Poor's (S&P) 500 Index, China Ming Sheng Bank (CMSB) and Dow Jones Industrial Average (DJIA) Index. The LSTM was used with a dropout layer to address the problem of overfitting. In order to further enhance the performance of single model, the LSTM was integrated with a series decomposition method Empirical Wavelet Transform (EWT), an error modeling technique Outlier Robust Extreme Learning Machine (ORELM) and an optimization algorithm Particle Swarm Optimization (PSO). The proposed hybrid model achieved better prediction accuracy than the benchmark models. Similar results were achieved in [30] with the LSTM units for temporal learning of Korean Composite Stock Index (KOSPI). Furthermore, an attention based encoder-decoder model was proposed in [19] to address the performance issues of RNNs for sequential data. Later on, the attention-based Transformer models have been widely used [31] and the use of attention layer has been effective in analyzing time series data [32]. For instance, a two-stage attention LSTM model was used for the day-ahead forecasts of the solar panels in [19]; the attention-based decoder was used prior LSTM for the extraction of input features and the attention-based encoder was applied post LSTM for capturing the temporal hidden patterns. A similar two-stage attention method was successfully applied in [33] to forecast the stock prices.

In a recent study, a novel optimization approach based on a Multi-Layer Sequential LSTM with adam optimizer has been used for stock prediction. The proposed model proved to be a high performer illustrating the training and testing accuracies of 96% and 98% respectively [28]. Likewise, another pertinent study [34] proposed a hybrid model of LSTM and relationwise graph attention network (ReGAT) for the trend prediction of cryptocurrencies' prices. The sequential patterns of individual cryptocurrency features were profiled by LSTM while ReGAT was used to extract the network features. The proposed model generated cryptocurrency portfolios with highest profits.

## 3 Problem statement

The standard finance theories like EMH and RWH are based on the over simplified assumptions of efficient markets, rational investors and information symmetry and they rule out the possibility of predicting stock markets on the basis of their past performance [21] and [22]. The proponents of these theories believe that any research effort predicting equity markets may not yield valid results and useful insights. However, the equity markets do not operate in ideal circumstances as posited by EMH and RWH, particularly for short to medium term duration. Alternatively, the adaptive market hypothesis combines the concepts of EMH and behavioral finance and argues that people are not always rational and behave according to the scenario and their motives [35]. Thus, there exist market inefficiencies due to the long-range dependence in the financial time series data [36]. There exists ample empirical evidence of the predictability of stock markets in the literature [3], [10], [13], [25], [26] and [28]. Our study also makes an effort to investigate the problem of stock markets' predictability using DL.

An insightful study of a stock market structure is not possible without considering many of the diversified factors which are directly or indirectly affecting the market. Modeling fewer technical indicators like in [26] and [30] limits the scope of the applied model by overlooking other important features. However, as many features of different nature (technical, economical, financial, political) are taken into account, some have more influence on the stock price than others. In this case, a suitable feature extraction method is needed without which the computational complexity of the applied model is low as in [26] and [13]. The choice of an appropriate dimensionality reduction algorithm is also important. For instance, a linear dimensionality reduction technique like Principal Component Analysis (PCA) cannot capture the non-linearities of stock prices' data [10].

Stocks prices are non-linear, non-stationary and noisy time series. Modeling complexity of such data without using a suitable signal decomposition method [17], [26] may yield compromised forecasts. The signal decomposition makes possible the exclusive analysis of a time series in the time domain and can enhance performance of the applied models [16].

The classical statistical methods like regression, ARMA, [4], ARIMA [5] etc. have been extensively used for predicting trend of stock markets. However, they are bound to common assumptions of linearity, normality and constant variance and their results are not applicable without these prior assumptions. While the financial time series data like stocks prices and returns are non-linear, skewed, and highly volatile and making them compliant to these statistical methods does not yield generalizable results [7]. Besides, many conventional ML techniques like DTs [17] and SVM [7] have short term dependencies and their performance is limited for modeling long-term trends of a stock market. These methods are also not suitable for a large data with many features. On the other hand, many NNs are prone to the problems of vanishing gradient and overfitting [37]. Inability of handling sequential data with long term dependency is another issue for the initial versions of NNs. Like, RNNs have short-term dependency due to their limited memory architecture [8].

The DL models used for stock predictions have various hyperparameters. The models' performance is linked to the values of these parameters, yet finding a set of best possible values for improved performance of the model is not a trivial task. Different optimization methods are used to tune a model's parameters boosting its learnability. In the absence of an optimization method [10], the learning algorithm is trained on hit and trial basis with a set of manually chosen hyperparameters limiting the scope of the model.

**Figure 1:**
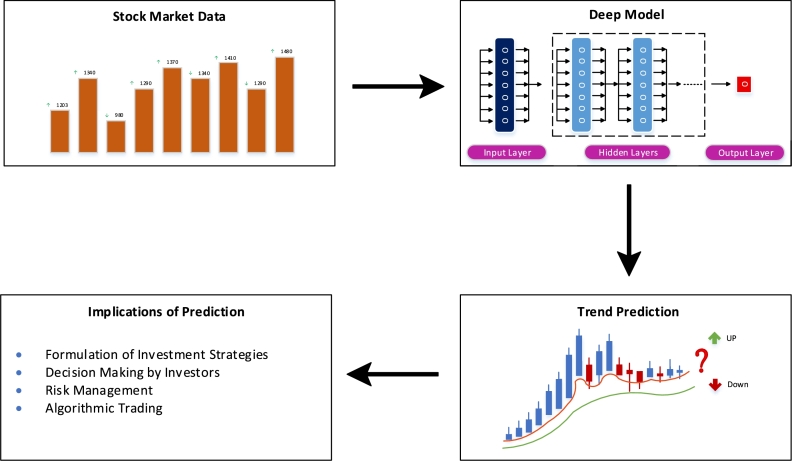
Implications of Predicting Stock Markets.

This study primarily focuses upon the robust prediction of stock markets using the non-linear, complex and noisy stock prices' data. The complexity and noise of the price series are addressed by using the time series decomposition technique EEMD. The dimensionality reduction method of CAE is chosen for extracting a set of significant features from the features space. Moreover, a DL based model PLSTM with temporal attention mechanism is proposed to address the problem of short-term dependencies of ML techniques for sequential data. The proposed model has several implications for investors in the stock markets as depicted in Fig. 1. A good prediction model with better performance facilitates investors in formulating more profitable investment strategies and better management of the inherent risk.

## 4 Proposed system model

**Figure 2:**
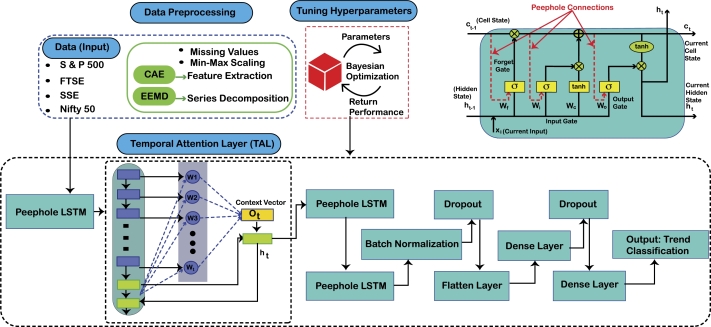
Proposed System Model PLSTM-TAL.

The prediction of stock markets is an important area of research for researchers, academicians, and investors. The investment strategies of institutional and individual investors are based upon these prediction models with the prime objective of earning profits and creating liquidity in the market. In this regard, this research work is an effort to analyze the sampled stock markets for proposing a better prediction model with enhanced accuracy. Hence, a recurrent DL prediction model the peephole LSTM with a TAL is proposed to efficiently predict the trend of four stock markets (U.S., U.K., India and China). Fig. 2 presents the architecture of the proposed system model. A recurrent architecture is proposed as recurrence is more appropriate for processing the complex sequential time series data. This is a novel hybrid model which has not been used earlier for financial time series prediction, as to the best of our knowledge.

An LSTM is a special RNN with gate mechanism and is better capable of processing sequential data and to capture both short and long-term dependencies in the time series. The forget and input gates allow the retention of useful historical information in the hidden and cell states. This retained information is further used for predicting the future data trends. The introduction of peephole in the LSTM architecture develops a linkage between the cell state and gates of LSTM providing access of already stored information in the cell state to the gates [38]. This enhances the prediction efficiency of the LSTM for sequential data with long-term patterns [39]. The hyperparameters of LSTM are fine-tuned with Bayesian optimization which is computationally more efficient than the random and grid search optimization methods as it also considers previous choices while optimizing a given set of parameters [20]. The final output of LSTM is then passed to the TAL which is introduced so that the special instances in the time series are given due attention before predicting the final trend of the stock market. Results of the proposed PLSTM-TAL have been compared with those of SVM, RF, CNN and LSTM (without feature extraction and series decomposition). The performance of all classification models have been validated using the evaluation metrics of accuracy, precision, recall, F1-score, Area Under the Receiver Operating Characteristics Curve (AUC-ROC), Area under the Precision Recall Curve (PR-AUC) and Matthew's Correlation Coefficient (MCC). The proposed hybrid model achieved higher performance scores than those of the benchmark models.

### 4.1 Data labelling

In this article, the trend classification of stocks' time series is binary, that is an upward trend if the return at a given time t+1 is greater than the return at time t [40]. Hence, the target variable $y_{i}(t)$ of the sample *i* at time *t* is given in equation (1) as follows:

(1) $$y_{i}(t)=\left\{ \begin{array}{cc} 1,\;if\;\; & r_{i}(t+1)>r_{i}(t),\\ 0,\; & Otherwise. \end{array}\right.$$

Here, $r_{i}(t+1)$ is the return at time t+1 whereas $r_{i}(t)$ is the return at time *t*. The class label is one for an upward trend and is zero for a downward trend.

### 4.2 Data preprocessing

At the foremost step, the open, high, low, close prices and volume of stocks' indices have been considered, and 40 technical indicators have been calculated from these OHLCV values. These are the same indicators as considered in [17]. The data is preprocessed for missing values and is then standardized using min-max scaling method. The dimensionality of the data has been reduced with contractive autoencoder and the extracted data contains the most pertinent features helpful for the enhanced performance of the proposed prediction model. The closing price time series have been denoised using the series decomposition algorithm of EEMD which is a noise assisted method that adds Gaussian noise to the original price series and then decomposes it into Intrinsic Mode Functions (IMFs). The IMF with higher complexity and least energy is subtracted from the original closing price generating a filtered time series. This filtered series is less complex and chaotic than the original series however, at the cost of losing some information. This filtered series along with other covariates of TIs have been used as input to the system model following the approach used in [17].

### 4.3 Contractive autoencoder

The contractive autoencoder is used for features extraction in this article. It was initially proposed in [18] as a regularized autoencoder which is used for learning representations for the subsequent classification tasks. The loss function of a traditional autoencoder is mathematically presented in equation (2). However, the CAE loss function is given in equation (3). The second term of this equation is the CAE regularization/penalty term that is expressed as the sum of squares of all the dimensional differential of the feature space. This regularization term is only applied to the training examples forcing the model to learn the salient patterns in the training dataset. Moreover, this term relates to the Frobenius norm of the Jacobian matrix of the encoder activations for the input. The use of Jacobian term enhances the locally invariant and robust encoding representations that suppresses the impact of noise speckles. Thus, the CAE is efficient for discrimination and robust feature representation. The inclusion of an explicit regularization term in the objective function is the exclusive property of the CAE making it robust to slight fluctuations in the input data and therefore it works well with financial data. This autoencoder is termed as ‘contractive’ as it contracts the dimensions of inputs neighborhood by mapping them to a smaller outputs' neighborhood. The process of dimensionality reduction with the CAE is presented in lines 5-11 of the Algorithm 2.

(2) $$||J_{h}(X)|{|}_{F}^{2}=\sum_{i}j{\left(\partial h_{j}(X)/\partial X_{i}\right)}^{2}$$

(3) $$Loss_{CAE}(\theta )=\sum (L(x,g(h(X))))+\lambda ||J_{h}(X)|{|}_{F}^{2}$$

### 4.4 Ensemble empirical mode decomposition

A time series is a combination of different harmonics the details of which can be studied using the frequency domain of signal analysis. Various methods like Fourier transformation and spectral analysis have been used for this purpose. However, filtering the signal for noise becomes very difficult especially when the fundamental harmonics are being shared by the noise [16]. Moreover, methods like Fourier transformation do not perform well for non-linear and non-stationary signals like stock prices. Therefore, an adaptive analysis tool EEMD proposed by Wu and Huang in [41] is used in this study for the decomposition of stock price signals. It is basically an extension of the Empirical Mode Decomposition (EMD) and was proposed to address the problem of mode mixing in EMD. It is an iterative method to decompose the given series into a set of orthogonal components called IMFs which are the quasi-stationary components of the original signal. The process of EEMD for sifting the original signal has following steps [10]:

Step 1: Add white Gaussian noise to the original series x(t) as: $x_{i}(t)=x(t)+wn_{i}(t)$ for i = 1, 2, 3, ..., n.

Here, $wn_{(}i)$ are the different realizations of the white Gaussian noise.

Step 2: Identify the local maxima and minima of the resultant time series $x_{i}(t)$.

Step 3: Create an upper envelope $e_{max}(t)$ for all local maxima and a lower envelope $e_{min}(t)$ for all local minima, using spline interpolation.

Step 4: Compute the average of the two envelopes as $m_{i}(t)=(e_{max}(t)+e_{min}(t))/2$

Step 5: Subtract this average from the data, $h_{i}(t)=x(t)-m_{i}(t)$. The $h_{i}(t)$ is an IMF ($c_{i}$) if it satisfies the following two conditions:

• The $h_{i}(t)$ is symmetric and may have only one extremum.
• Its mean is approximately zero.

Step 6: If $h_{i}(t)$ does not meet the above two conditions, then it replaces the original series $x_{(}t)$ and steps 1-6 are repeated until it meets the criteria of being an IMF.

Step 7: When $h_{i}(t)$ is an IMF ($c_{i}(t)$), then the residual signal in $x(t)=h_{i}(t)+r_{i}(t)$ is then passed through the same sifting procedure until it becomes a monotonic function from which no more IMFs can be extracted.

Finally, the IMFs can be summed to get the final series with the residue as: $X_{t}=\sum_{n=1}^{N}c_{i}(t)+r_{N}(t)$

The process of EEMD is presented in Algorithm 1. The input, output and the variables are given in lines 1-4 and lines 5-21 present the decomposition process of the price series. It is pertinent to mention that the IMFs of a signal can be modeled and predicted independently and then the resultant predictions are reconstructed to get a final prediction. However, the accumulative effect of the prediction errors of sub-signals aggravates the final prediction error. Therefore, a filtered series is generated in this study by subtracting the most complex and chaotic IMF ($C_{1}$) from the original series. The Sample Entropy (SaEn) value is used as a proxy for the complexity of IMFs presented by their noise level. The noise level of each IMF is calculated as per the lines 22-32 of the Algorithm 1. Afterwards, only the filtered series is predicted by the proposed model. This is the same approach as followed in [16].

**Algorithm 1:**
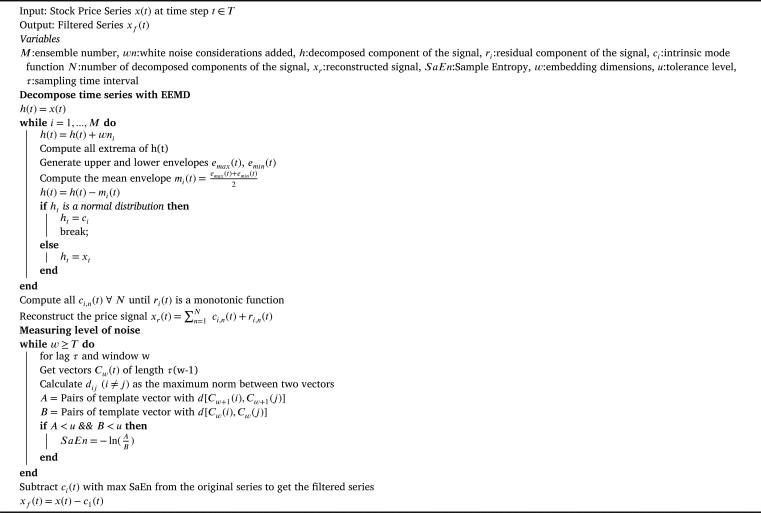
Filtering Stock Price Signals for Noise using EEMD and SaEn

### 4.5 Long short term memory

RNNs were developed to overcome the limitations of ANNs in dealing with sequential data like text and images etc. They have been successfully used in speech recognition and natural language processing tasks. Their main purpose was to predict the next probable outcome by using the order in the data. However, they could only remember few recent orders or patterns because of their short-term memory structures and therefore they cannot relate older data patterns with current ones. Moreover, they are prone to the vanishing gradient problem [42]. In order to overcome these limitations of RNNs, Hochreiter and Schmidhuber in [43] developed a special version of RNNs as LSTM network by introducing a memory line the Constant Error Carrousel (CEC). The LSTM derives its name from its structure that helps in developing long-term memory (the cell state) with the help of short-term memory (the hidden state). In simpler words, it is an artificial neural network with feedback connections capable of processing not only single data points but entire sequences of the data. The feedback mechanism of an LSTM is comprised of the CEC and the three gates (forget, input and output). The cell retains information over arbitrary long-term intervals while the gates control the flow of information by keeping or forgetting it. The information is filtered on the basis of its relevance and importance to predict the future values. An LSTM is a viable deep network solution for time series data which may have time lags of indefinite duration between important events.

In a recent study, a novel optimization approach based on a multi-layered sequential LSTM with adam optimizer has been used for stock prediction. The proposed model illustrated the training and testing accuracies of 96% and 98% respectively thus outperforming other ML and DL algorithms [28]. In another work [6], the stock markets' time series have been forecasted with dual-LSTM using the sequentially moving window approach. The dual-LSTM outperformed all benchmark models and showed that RNN based models can forecast better than the classical models like ARIMA.

### 4.6 Peephole LSTM

One of the limitations of the traditional LSTM is the inability of its gates to access the output of the memory unit when the output gate is closed. Moreover, the learning of precise timings of fluctuations in a time series is not possible unless the memory cell is allowed to control the gates and that is only possible with an open output gate. To address this problem, Gers and Schmidhuber in [38] proposed an LSTM architecture with a peephole providing access of the cell state to the gates of LSTM even when the output gate is closed. With the peephole, the gates of the LSTM can access the cell state even when the output gate is closed [39]. Therefore, the forget, input and output gates of the LSTM consider the cell state as input and are updated accordingly as presented in the equations (4) - (10). However, the only difference of peephole LSTM from that of the original LSTM is the calculation of additional connections, while the rest of the working principle is same. The LSTM gates can access the CEC with the peephole connections.

(4) $$f_{t}=\sigma_{z}(W_{f}.[C_{t-1},h_{t-1},x_{t}]+b_{f})$$

(5) $$i_{t}=\sigma_{z}(W_{i}.[C_{t-1},h_{t-1},x_{t}]+b_{i})$$

(6) $${\overset{ˆ}{c}}_{t}=\sigma_{z}(W_{c}x_{t}+U_{c}h_{t-1}+b_{c}),$$

(7) $$c_{t}=f_{t}\;⁎\;c_{t-1}+i_{t}\;⁎\;{\overset{ˆ}{c}}_{t},$$

(8) $$o_{t}=\sigma_{z}(W_{o}.[C_{t},h_{t-1},x_{t}]+b_{o})$$

(9) $$h_{t}=o_{t}\;⁎\;\sigma_{z}(c_{t}).$$

(10) $$y_{t}=h_{t}$$

In the above PLSTM equations, $f_{t},i_{t},o_{t},c_{t},h_{t}$ and ${\overset{ˆ}{c}}_{t}$, represent output of the forget gate, input gate, output gate, cell state, hidden state and predicted value of the cell state respectively. Moreover, $x_{t}$ represents the input data at time t; $C_{t-1}$ and $h_{t-1}$ are the cell and hidden states at time t-1; $W_{f},W_{i},W_{o}$ and $W_{c}$ represent the weight matrices of the forget gate, input gate, output gate and the cell state and $b_{f},b_{i},b_{o},b_{c}$ are the offsets (bias terms) respectively. $\sigma_{z}$ is the sigmoid activation function. Other activation functions like tanh can also be used. Lastly, $y_{t}$ is the final output (label).

The connection of LSTM gates with $C_{t-1}$ significantly enhances the accuracy of sequence-to-sequence prediction tasks. The peephole convolutional LSTM has been used to extract abstractive summaries [44], predict wind speed [39], for electricity load prediction [45] and for theft detection in smart grid [46]. However, the usefulness of the peephole LSTM has not been proved in some studies [45]. This study considers peephole LSTM in lieu of simple LSTM as the former is more appropriate for long-term sequence forecasting problems. The detailed algorithm for the proposed PLSTM-TAL is given in Algorithm 2. Lines 1-4 describe the input space, output and the variables. The working of peephole LSTM with TAL is presented in lines 12-30 of the same algorithm.

**Algorithm 2:**
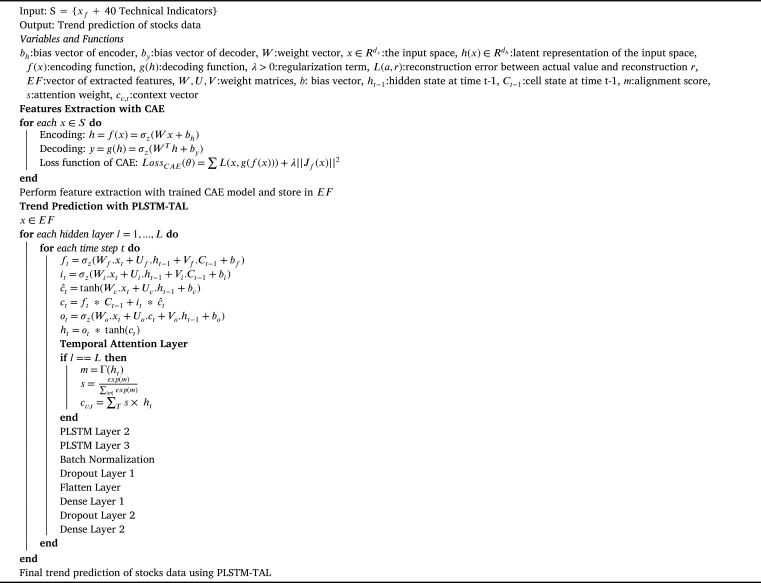
The Proposed Model PLSTM-TAL

### 4.7 Bayesian optimization

The utility of LSTM for a particular problem depends upon the values of its hyperparameters. For instance, size of the time window (input) contains contextual information which is important for forecasting price trends. A small input window is usually deficient of significant signals while a large window is often noisy with extra information. Moreover, number of hidden layers and number of neurons in those layers decide upon the computational power of the model. While, many studies applied LSTM with manual selection of hyperparameters, others applied some heuristics algorithms for optimization [30]. In this study, the bayesian optimization method for optimization of LSTM hyperparameters is applied. Bayesian method was proposed by Jonas Mockus in [47] as a sequential method for global optimization of black-box models. The bayesian method converges to the optimal solution in fewer iterations and performs better than other optimization techniques like random search and grid search [20]. Unlike random and grid search methods, bayesian algorithm speeds up the optimization process while considering the previous performances. Whereas, grid and random search methods perform independent of their previous evaluations. Thus, bayesian provides more efficient optimization as it converges to the optimized solution in fewer iterations [48]. The optimized values of the hyperparameters of our proposed model are given in Table 2.

**Table 2:** Tuning of PLSTM-TAL Hyperparameters using Bayesian Optimization.

Hyperparameters	Range of values	Optimal value
Units	16, 32, 64, 128	64
Activation Function	relu, tanh, linear, sigmoid	tanh
Optimizer	SGD, Adam, Adamax, RMSprop, Adagard	Adamax
Loss	binary crossentropy, hinge, square hinge	binary crossentropy
Dropout	0.1, 0.2, 0.3, 0.4	0.1

### 4.8 Attention layer

A feed-forward NN considers all input features as unique and independent implying that nothing can be inferred from the current feature about the next consecutive feature. This approach is suitable for a data having no association and dependencies among variables. However, it is not viable in the presence of an underlying local structure to the data. Besides, the DL models like RNNs consider an input as a long data string and generate an output of shorter length thus losing some information. RNNs have to develop connections between lengthy input and output sequences with dozens of words. RNNs complemented with attention mechanism are capable of predicting a particular output sequence by focusing on certain parts of the input sequence. LSTMs like other advanced models have their limitations specially when dealing with long data sequences. LSTM based encoder-decoder networks have been used for time-series forecasting purposes, particularly when sequence-to-sequency mapping is required. These models have performed exceptionally well with small sequences however their performance declines with longer sequences [19] and [49]. Moreover, LSTMs can circumvent the vanishing gradient problem [50], yet they are sensitive to the exploding gradient issue. In textual data analysis, LSTMs like other RNNs, give higher weights to words in closer proximities and the upstream context is emphasized higher than the downstream context. The attention mechanism is capable of addressing the aforementioned limitations. The attention mechanism was proposed by Bahdanau et al. in [49] that is a three steps process to compute alignment scores, weights and context vector. It was introduced to increase the performance of ML tasks using encoder-decoder models. Our brain does not consider and process the whole set of overwhelming background information, rather it picks and processes only the important information for the task at hand and discards the rest of it rendering it as irrelevant. This feature of brain is termed as its ‘attention mechanism’. The same can be incorporated in NNs. The NNs with attention principle are adept to comprehend the sequential data like textual, video, voice or time series. Main purpose of attention is to filter the data for important sequences be them spatial or temporal. It compares current inputs with the previously stored ones. The attention-based NNs are computationally faster than the RNNs and LSTMs in capturing the time dependent context-based patterns from the data. This study uses attention layer in the proposed model because the data of stocks indices is time dependent and contextual. Besides making accurate predictions, the attention LSTM also helps the researchers in understanding the reasons for these predictions by providing intermediate outputs. In [19], the authors applied attention layer to the inputs focusing more on relevant features with respect to time and then they integrated a TAL to emphasize on the relevant temporal hidden states of the LSTM units. The attention layer is integrated at the output of the hidden states of the proposed LSTM model.

## 5 Simulations and results

This study considers the data of stock indices of S&P 500, the Financial Times Stock Exchange (FTSE), Shanghai Stock Exchange (SSE) Composite and Nifty 50 representing the equity markets of United States (U.S.), United Kingdom (U.K.), China and India respectively. The purpose is to compare the two developed and leading stock markets of the world with the two emerging markets. The U.S. and U.K. indices represent the developed markets while China and India are representing emerging markets. The daily data of these four indices were considered for a time span of 17 years and three months (January 01, 2005 – March 31, 2022). This is a large time span inclusive of the impact of 2008 global financial crisis and the COVID-19 pandemic. The S&P 500 is the most followed index as it tracks the performance of the top 500 companies in the U.S. equity market. Top nine companies in terms of market capitalization on its list are Apple, Microsoft, Alphabet, Amazon, Meta Platforms, Tesla, Nvidia, Berkshire Hathaway and JPMorgan Chase. These companies accounted for 28.1% of the market capitalization of S&P 500 as of September 30, 2021. On the other hand, FTSE represents the 100 companies of the London Stock Exchange (LSE) with the highest market capitalization. The stock markets of U.S. and U.K. account for approximately 70% of the Morgan Stanley Capital International (MSCI) Developed Markets Index. Besides, the stock markets of China and India account for around 44% of the MSCI Emerging Markets Index.

**Figure 3:**
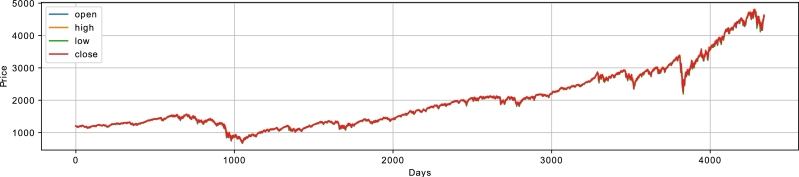
S&P500 Index.

The pricing trend of the S&P 500 index is illustrated in Fig. 3. From the figure, it is observed that after three years of the considered period, there is a decline in the S&P 500 index during 2008-2009. This decline is because of the well-known financial downturn of 2008 in U.S. which was primarily caused by the sub-prime mortgage crisis of 2007. The U.S. stock market did not sufficiently recover until mid-2013 [51]. Another dip in the index is observed during March 2020 due to the worldwide spread of COVID-19 and worsening situation in U.S. However, this impact was not for long as the market started to recover in April 2020 and followed an upward trend. Similar trends are observed in the price indices of FTSE, SSE and India. These datasets have high non-linearity, randomness and chaotic behavior, which becomes challenging for classical statistical models to forecast. Initially, the datasets contain Open, High, Low, Close, and Volume (OHLCV) as five features. Afterwards, forty technical indicators as used in [17] are computed from OHLCV and included as features to increase the prediction performance of the proposed model. Subsequently, extensive simulations are performed on the datasets. The discussion about simulation results is provided in the next subsection.

### 5.1 Results and discussion

**Figure 4:**
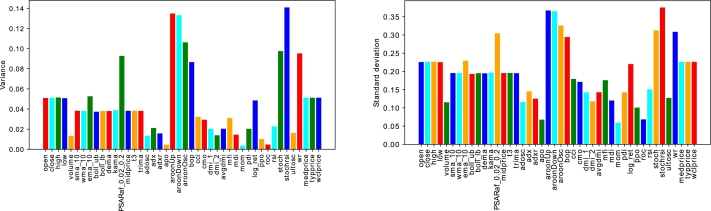
(a) Variance of Different Features of S&P500 (b) Standard Deviation of Different Features of S&P500.

An exploratory data analysis of the indices is performed to analyze their behavior. Standard deviation and variance of the datasets are calculated to check the spread of features around their mean values. Fig. 4(a) and Fig. 4(b) respectively demonstrate the level of variance and standard deviation between the features of the S&P 500 dataset. Both standard deviation and variance are the measures of spread. Standard deviation measures how a group or a cluster is far away from the central tendency, while variance measures how much each element differs from the mean point.

**Figure 5:**
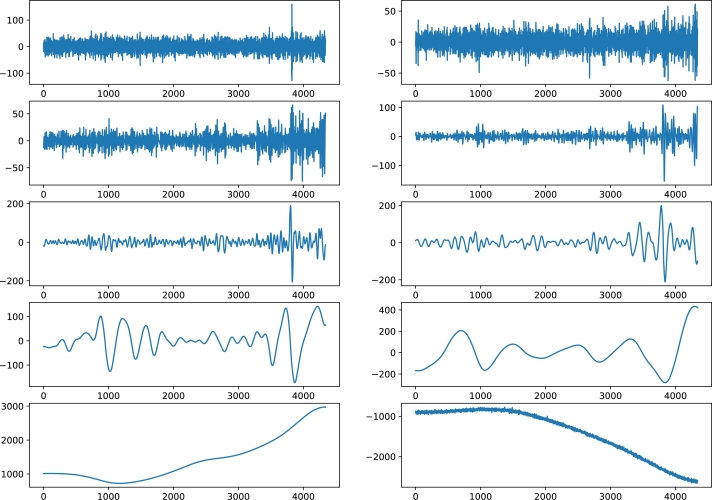
EEMD based Decomposition of S&P500 Index - IMF_1_ to IMF_9_ and the Residual Component at the end.

**Figure 6:**
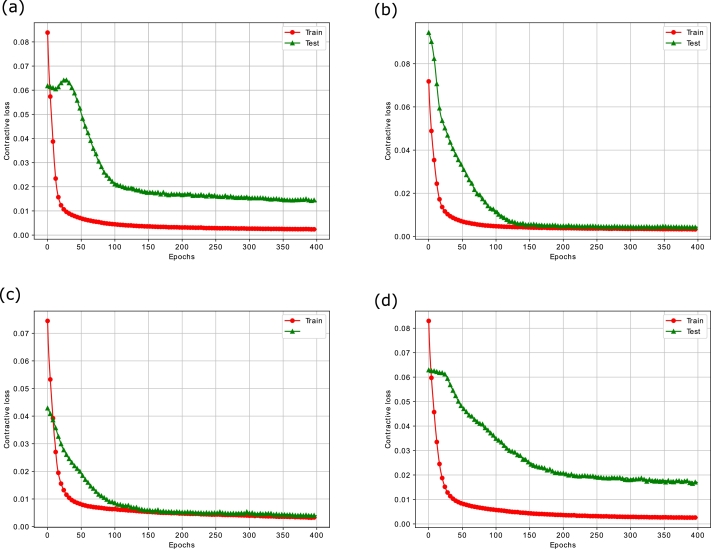
(a) Contractive Loss of CAE on S&P500 (b) Contractive Loss of CAE on FTSE (c) Contractive Loss of CAE on SSE (d) Contractive Loss of CAE on Nifty.

It is evident that the variance of around 30 features is less than 0.04 implying the need for extraction of features explaining the maximum variability in the data. For this purpose, the contractive autoencoder is used. Moreover, the presence of randomness and noise in the stock data is another factor, which adversely affects the performance of the supervised learning models. The removal of noise is necessary for an efficient analysis of the time series data. In this regard, ensemble empirical mode decomposition (EEMD) is applied to denoise the closing price of the four stock indices. The EEMD technique decomposes the original time series signal into multiple Intrinsic Mode Functions (IMFs), as shown in Fig. 5. From the figure, we analyze that the initial IMFs have greater amplitude and frequency, which show a high level of non-linearity and randomness. Whereas, the subsequent IMFs and residue follow the linear relationship between features and have a low ratio of noise. Furthermore, we use sample entropy to calculate the noise and complexity of each IMF, as given in Table 3. The notable thing is that the top most IMFs have a high value of entropy. Therefore, we remove the first IMF with the highest sample entropy from the original series to denoise the stock data. Afterwards, a Contractive Autoencoder (CAE) is employed for the efficient extraction of the prominent and significant features. The bar charts of Fig. 4(a) and Fig. 4 (b) show that many features have high values of standard deviation and variance indicating high level of volatility in the data. The convergence of contractive loss for S&P 500, FTSE, SSE and Nifty indices are shown in Fig. 6(a) to 6(d) respectively

**Table 3:** Sample Entropy Values for Decomposed Components of S&P500 Index.

Decomposed Series	Sample Entropy (Complexity)
Original series (x)	4.2485
IMF1	4.2195
IMF2	3.7376
IMF3	2.5359
IMF4	1.3784
IMF5	0.7362
IMF6	0.6112
IMF7	0.5165
IMF8	0.2560
IMF9	0.0857
Residue	2.5649

It is seen that the loss value decreases at each epoch, which indicates that the CAE efficiently learns the hidden non-linearities from the markets' data. Furthermore, the integration of Frobenius norm in the loss function of CAE improves its convergence speed and makes it robust against granular changes in the stock patterns. In addition, the declining loss in both training and testing phases affirms that the CAE model intelligently grasps the stock patterns and yields a compressed representation of the feature space accordingly.

**Figure 7:**
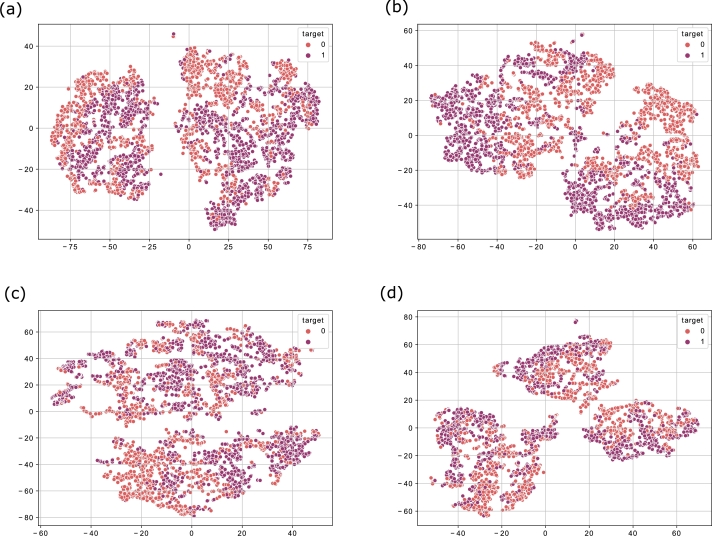
(a) CAE Extracted Features for S&P500 (b) CAE Extracted Features for FTSE (c) CAE Extracted Features for SSE (d) CAE Extracted Features for Nifty.

After the successful training of the CAE, the encoder model is applied for the extraction of plausible features. Fig. 7 (a) to (d) shows the CAE extracted features for the four stock indices. From the figure, it is observed that the selected features of CAE are the most plausible features as they are not overlapping with each other. This also implies that each feature has high variance and separate impact. Thereby, the extracted feature set is passed to the PLSTM-TAL model for the final prediction of the stock market movement.

**Figure 8:**
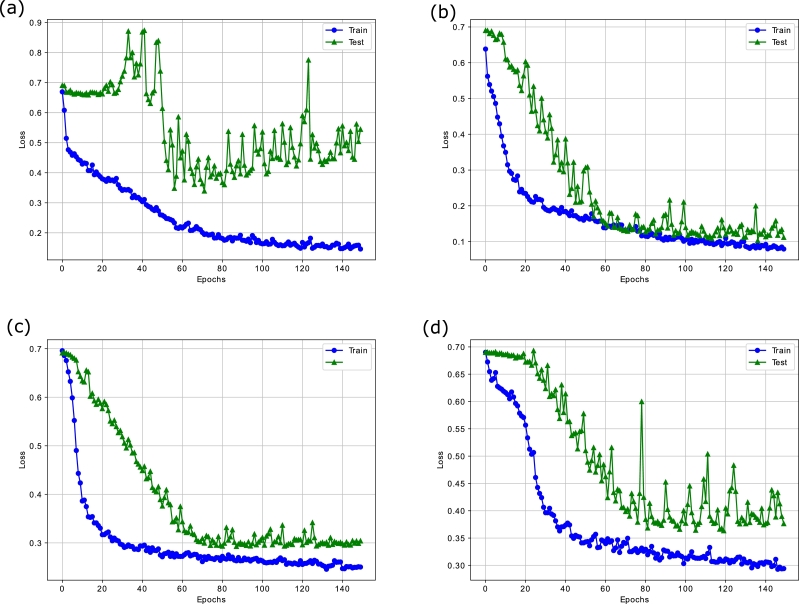
(a) Loss Analysis of PLSTM-TAL on S&P500 (b) Loss Analysis of PLSTM-TAL on FTSE (c) Loss Analysis of PLSTM-TAL on SSE (d) Loss Analysis of PLSTM-TAL on Nifty.

**Figure 9:**
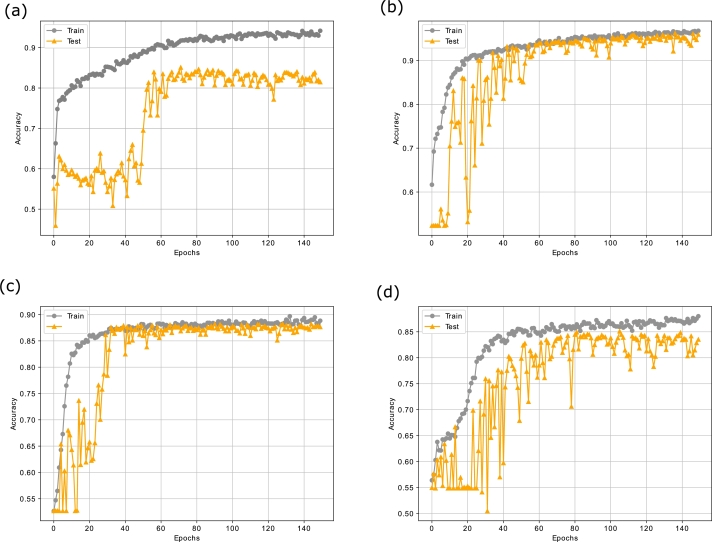
(a) Accuracy Analysis of PLSTM-TAL on S&P500 (b) Accuracy Analysis of PLSTM-TAL on FTSE (c) Accuracy Analysis of PLSTM-TAL on SSE (d) Accuracy Analysis of PLSTM-TAL on Nifty.

The convergence analyses of the proposed PLSTM-TAL model in terms of loss and accuracy are depicted in Figure 8, Figure 9 respectively. From Figs. 8(a), 8(b), 8(c), 8(d), it is noted that there are fewer fluctuations in the training loss than the testing loss. The reason is that the testing data is unseen and the model struggles to capture the complex non-linear patterns of the stock markets' data. That is why the model provides lower granular feedback on the testing set. Moreover, more fluctuations in the testing loss are seen in Figs. 8(a) and 8(d) as compared to 8(b) and 8(c). This is because that the datasets of S&P 500 and Nifty 50 have more ups and downs as compared to those of FTSE and SSE. Therefore, the proposed model gets confused on some unseen patterns of these datasets. However, it still performs better as compared to the benchmark models.

**Figure 10:**
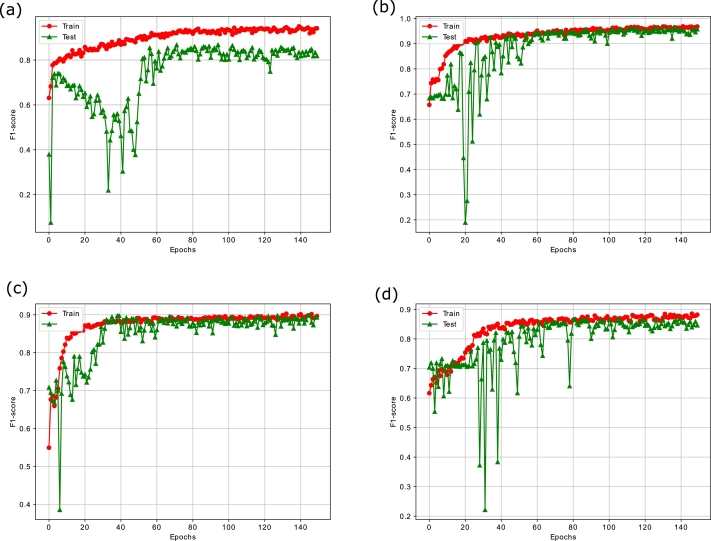
(a) F1-score Analysis of PLSTM-TAL on S&P500 (b) F1-score Analysis of PLSTM-TAL on FTSE (c) F1-score Analysis of PLSTM-TAL on SSE (d) F1-score Analysis of PLSTM-TAL on Nifty.

**Figure 11:**
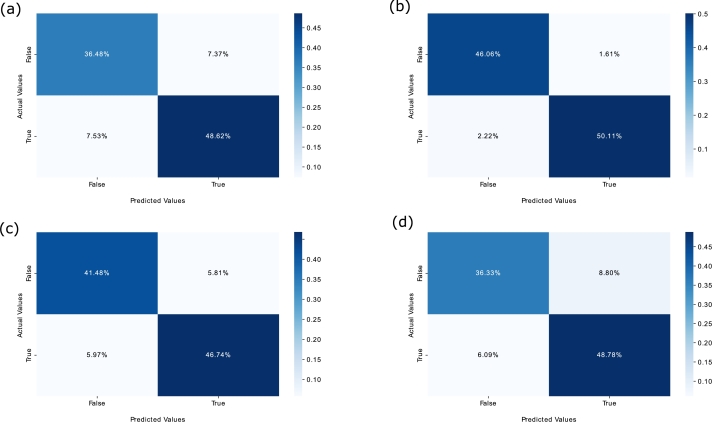
(a) Confusion Matrix of PLSTM-TAL on S&P500 (b) Confusion Matrix of PLSTM-TAL on FTSE (c) Confusion Matrix of PLSTM-TAL on SSE (d) Confusion Matrix of PLSTM-TAL on Nifty.

Similarly, Figs. 9(a), 9(b), 9(c), 9(d) show the epoch wise accuracy analysis of the proposed model for all datasets. It is observed that the training accuracy is smooth while there are fluctuations in the validation accuracy, yet the model converges well towards an optimal solution. Besides, the F1-score analysis of the proposed model for all the datasets is illustrated in Figs. 10(a), 10(b), 10(c), and 10(d). The F1-score basically measures the balance ratio of precision and recall. From the figures, it is observed that the model achieves good results except for some occasional ups and downs in the validation set. The reason is that the stock markets' data is highly non-linear and chaotic, still the model converges well due to the integration of the temporal attention mechanism and obtains a suitable F1-scores of 0.8671, 0.9632, 0.8880 and 0.8676 on the validation sets of S&P 500, FTSE, SSE and Nifty respectively. Moreover, Figs. 11(a), 11(b), 11(c), 11(d) exhibit the confusion matrix of the proposed PLSTM-TAL model depicting the percentage of true and false predictions for the unseen portions of all datasets. These figures imply that the correct predictions for S&P 500, FTSE, SSE and Nifty 50 are 85.30%, 96.17%, 88.82% and 85.11% respectively. This implies that datasets of FTSE and SSE have been more correctly classified than those of S&P 500 and Nifty 50. This is in line with the previous analyses of loss and accuracy convergence for the same datasets.

**Figure 12:**
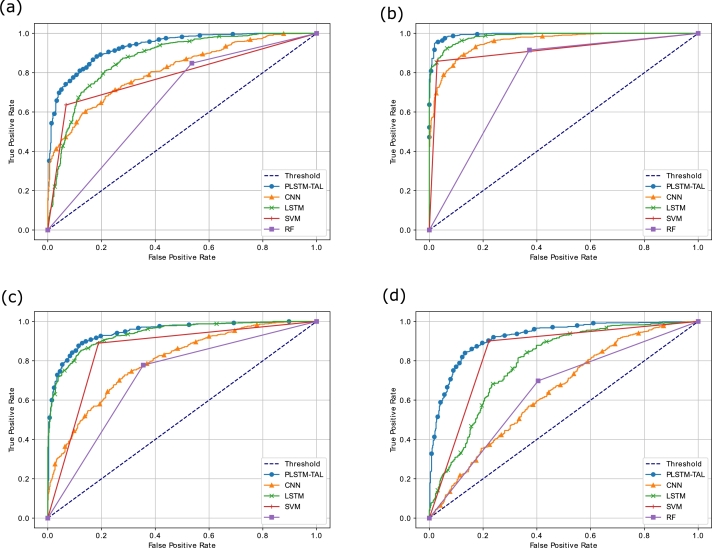
(a) AUC-ROC of the Proposed and Benchmark Models on S&P500 (b) AUC-ROC of the Proposed and Benchmark Models on FTSE (c) AUC-ROC of the Proposed and Benchmark Models on SSE (d) AUC-ROC of the Proposed and Benchmark Models on Nifty.

**Figure 13:**
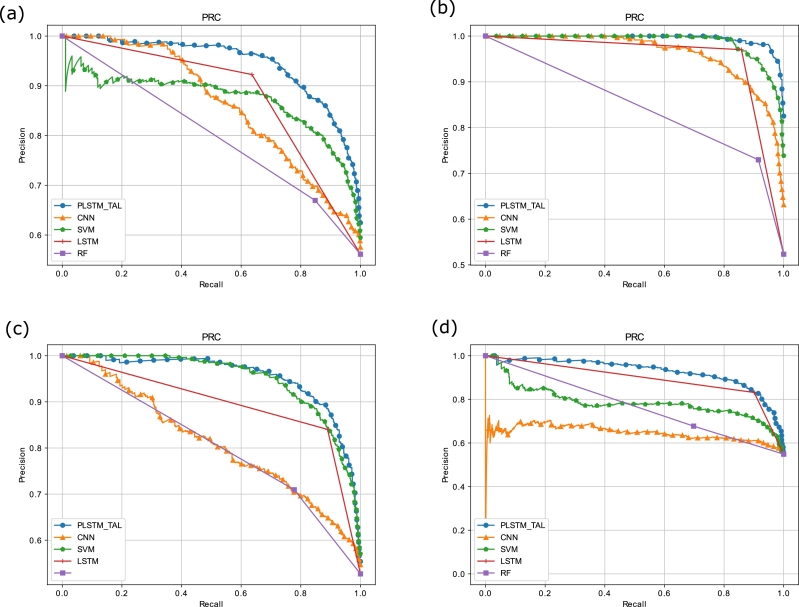
(a) PR-AUC of the Proposed and Benchmark Models on S&P500 (b) PR-AUC of the Proposed and Benchmark Models on FTSE (c) PR-AUC of the Proposed and Benchmark Models on SSE (d) PR-AUC of the Proposed and Benchmark Models on Nifty.

The AUC-ROC curves for all the datasets are shown in Figs. 12(a), 12(b), 12(c), 12(d). Among all the benchmark models, our proposed model gains the maximum value of AUC-ROC of 0.9312, 0.99942, 0.9464, and 0.9195 on S&P 500, FTSE, SSE and Nifty 50 indices, respectively. A high value of AUC-ROC indicates that the PLSTM-TAL model differentiates well the samples of upward and downward trends. Using this metric, the values of FPR and TPR are computed for the models at different classification thresholds. From the figure, it is clearly seen that the area under the ROC is maximum for the proposed model for the four indices followed by LSTM for first three indices and SVM for Nifty 50. The top left corners of the Figs. 11(a) to 11(d) show that the proposed model successfully maintains the peak value. Thus, the integration of the peephole connections and the TAL in the proposed model enable it to learn the non-linear and long-term temporal patterns of the stock markets' data in an efficient manner. Lastly, Figs. 13(a), 13(b), 13(c), 13(d) illustrate PR-AUC of the proposed and the baseline models on the validation sets of all indices. The primary purpose of this metric is to measure the balanced ratio of precision and recall at different classification thresholds. The precision score represents the Positive Predictive Value (PPV) and tells the percentage of correct positive predictions out of total positive predictions made by the proposed model. Whereas, recall represents how many correct positive class predictions are found from the actual number of positive class. The proposed model obtains highest PR-AUC values of 0.9455, 0.9948, 0.9539 and 0.9281 on S&P500, FTSE, SSE and Nifty respectively thus beating the benchmark models. The top right peak of the PR-AUC curve shows its highest value and our proposed model maintains the peak value for all indices followed by LSTM and SVM. Therefore, it is concluded that the proposed model is an efficient solution to predict the trend of the sampled stock markets. Moreover, the performance metrics of the proposed model PLSTM-TAL are compared with those of the benchmark models (CNN, LSTM, SVM and RF) and their values are presented in Table 4, Table 5, Table 6, Table 7 for all the datasets. These tables show that the proposed model outperforms the benchmark models in terms of accuracy, F1-score, MCC, AUC-ROC and PR-AUC. The probable reason is that the proposed model leverages the benefits of both peephole connections and the temporal attention mechanism. Moreover, the hybrid models are expected to overperform the individual classification models.

**Table 4:** Performance Metrics of the Proposed and Benchmark Models for S&P500.

Schemes	Accuracy	Precision	Recall	F1-score	MCC	AUC-ROC	PR-AUC
PLSTM-TAL	0.8509	0.8683	0.8659	0.8671	0.6975	0.9312	0.9455
CNN	0.7089	0.7009	0.8399	0.7641	0.4030	0.8111	0.8628
LSTM	0.8049	0.8015	0.8673	0.8331	0.6019	0.8720	0.8704
SVM	0.7657	0.9226	0.6361	0.7530	0.5784	0.7839	0.8815
RF	0.6797	0.6695	0.8481	0.7483	0.3419	0.6561	0.8014

**Table 5:** Performance Metrics of the Proposed and Benchmark Models for FTSE.

Schemes	Accuracy	Precision	Recall	F1-score	MCC	AUC-ROC	PR-AUC
PLSTM-TAL	0.9617	0.9689	0.9576	0.9632	0.9234	0.9942	0.9948
CNN	0.8791	0.8451	0.9415	0.8907	0.7618	0.9569	0.9622
LSTM	0.9273	0.9201	0.9429	0.9314	0.8544	0.9847	0.9868
SVM	0.9135	0.9703	0.8611	0.9125	0.8330	0.9161	0.9520
RF	0.7781	0.7296	0.9152	0.8119	0.5709	0.7714	0.8446

**Table 6:** Performance Metrics of the Proposed and Benchmark Models for SSE.

Schemes	Accuracy	Precision	Recall	F1-score	MCC	AUC-ROC	PR-AUC
PLSTM-TAL	0.8821	0.8894	0.8867	0.8880	0.7637	0.9464	0.9539
CNN	0.6903	0.6525	0.8822	0.7501	0.3956	0.7935	0.8123
LSTM	0.8622	0.8850	0.8489	0.8666	0.7250	0.9375	0.9485
SVM	0.8527	0.8402	0.8897	0.8642	0.7049	0.8506	0.8940
RF	0.7149	0.7093	0.7779	0.7421	0.4273	0.7113	0.8021

**Table 7:** Performance Metrics of the Proposed and Benchmark Models for Nifty.

Schemes	Accuracy	Precision	Recall	F1-score	MCC	AUC-ROC	PR-AUC
PLSTM-TAL	0.8511	0.8471	0.8891	0.8676	0.6988	0.9195	0.9281
CNN	0.6254	0.6223	0.8071	0.7028	0.2326	0.6429	0.6457
LSTM	0.7528	0.7280	0.8771	0.7956	0.5033	0.7901	0.7869
SVM	0.8455	0.8315	0.9010	0.8648	0.6882	0.8395	0.8934
RF	0.6517	0.6771	0.6979	0.6874	0.2945	0.6467	0.7704

#### 5.1.1 Significant findings of the study

The experimental results show the superior performance of our proposed model for all datasets followed by LSTM and SVM. PLSTM-TAL achieves the highest accuracy, F1-score, MCC, AUC-ROC and PR-AUC for all stock markets. Moreover, the methods of EEMD, CAE and Bayesian optimization enhance the performance of the proposed PLSTM-TAL. The accuracy of the proposed model is 96% and 88% for U.K. and Chinese stock markets, respectively and it is 85% for both U.S. and Indian markets. Hence, the stock markets of U.K. and China are found to be more predictable than those of U.S. and India. Moreover, the TAL significantly improves the prediction performance of the proposed model by enabling it to focus on the relevant part of the input sequences. PLSTM with TAL can better identify the long-term dependence and temporal patterns in the data of analyzed stock markets. Finally, our proposed model can be used to formulate profitable and timely trading strategies in the equity markets.

#### 5.1.2 Limitations of the study

Our study has certain limitations. First, the daily data of stock indices is used which is deficient of the details for intra-day fluctuations of prices. It is difficult for day traders to formulate their trading strategies without analyzing intra-day data. Second, other methods of features' extraction and hyperparameters' optimization have not been contrasted with the applied models of CAE and Bayesian optimization, respectively. Third, the impact of social and electronic media, political uncertainty and geographical risk on the stock markets have not been considered. Equity markets are not isolated from these effects. In this regard, indicators like Twitter-based Economic Uncertainty (TEU), Economic Policy Uncertainty (EPU) and Geopolitical Risk (GPR) can also be considered by the future prediction models. Future studies can extend the applications of the proposed model addressing these limitations.

## 6 Conclusion

Stock prices in a stock market of a smart city are fluctuated due to many diversified factors of both internal and external environments. It is difficult to reveal the inherent rules of price fluctuations of a complicated non-linear stock market using the traditional prediction methods. This paper proposes a novel DL model PLSTM-TAL to efficiently predict the future direction of the sampled stock markets. The daily data of four stock indices viz. S&P 500, FTSE, SSE and Nifty from January 2005 to March 2022 was analyzed. In order to validate the robustness of the proposed model, various simulations have been performed. The proposed model is also compared with the DL models CNN and LSTM and ML techniques SVM and RF using seven performance metrics of accuracy, precision, recall, F1-score, MCC, AUC-ROC and PR-AUC. The proposed model outperforms the benchmark models for all the datasets having highest scores for most of the performance metrics. Highest scores of the proposed model for AUC-ROC and PR-AUC prove its ability to differentiate well among downward and upward trends. The results provide empirical evidence for robustness of the proposed model in capturing the complex hidden patterns in the observed data. The role of peephole in the LSTM architecture is significant in enhancing the efficiency of LSTM. Besides, the attention layer complemented the PLSTM performance for identifying the inherent dynamics of price fluctuations with respect to time. The equity markets of U.K. and China have found to be more predictable than those of U.S. and India. One probable reason may be that the former markets are more stable than the later ones. Another debatable argument may be derived from the classical finance theories that the stock markets of U.S. and India are more efficient and therefore less predictable [21] and [22].

### 6.1 Future work

Our proposed model presented in this study can be further enhanced in the future in the following suggested ways. These may add to the generalizability and interpretability of our proposed model and hence can be significant research contributions for predicting stock markets.

#### 6.1.1 Use of higher frequency data

The proposed model can be trained with higher frequency data instead of daily prices. With the advancements of technology, the wider availability and access to higher frequency data has enabled researchers to investigate the issues of stock markets which could not be studied earlier due to the non-availability of such data. Forecasting stock markets' intra-day information is also important for algorithmic trading where hourly, per minute and even per second data are analyzed for generating sell and buy signals. The research in the field of finance has considerably evolved around high-frequency domain in recent decades and remains challenging due to several issues. The modification of the existing ML and DL models and the development of new methods for their compatibility with the finer data frequencies are a few to mention. Future research involving the modification of our proposed model for higher frequency stock markets' data can be insightful.

#### 6.1.2 Inclusion of other factors

The price changes in modern stock markets are influenced by many diverse factors from the external environment – economic, geopolitical, social media, local and global uncertainties, etc. In this scenario, the study can be made more comprehensive with the inclusion of some domestic and global uncertainty factors like EPU, GPR and Financial Stress Index (FSI). The effect of changes in monetary policy on the stock market can also be assessed including indicators like Shadow Short Rate (SSR). Moreover, TEU, pandemic scenario analysis, and Google and Wikipedia Search trends are the other important contemporary factors deriving the modern equity markets. Therefore, including them in the feature space may provide us with more insightful predictability analysis of the markets. The study of stock markets with the inclusion of the aforementioned inputs is the potential area that needs more attention and exploration.

#### 6.1.3 Comparison with other methods of dimensionality reduction and optimization

Higher prediction accuracy becomes challenging in the presence of a variety of features impacting prices in stock markets. Features' extraction is important to reduce irrelevant variables and computational cost, and improves the ML models' performance. Hence, this work can also be extended in future by including a comprehensive comparison of the performance of the proposed model with different dimensionality reduction techniques. For example, PCA, RF and embedded methods like LASSO and Ridge regression can be used for feature extraction along with CAE and the best performing method can be selected for the proposed deep model. Important features can also be selected based on Spearman Correlation Analysis (SCA) as in [52].

Similarly, future research works can hyper-tune the proposed model with some meta-heuristic techniques like genetic and evolutionary algorithms, mathematical optimization, grey wolf optimization, etc. Meta-heuristic techniques are more appropriate for optimizing complex and non-linear problems in minimum time [53]. This is important and may be a valuable addition to our proposed model as some better optimization technique other than Bayesian optimization can better-tune the hyperparameters of PLSTM-TAL.

#### 6.1.4 Model's robustness

Another prospective improvement can be that the robustness of the proposed model is further tested and validated using a sliding window cross-validation method or adding some noise to the test data series. Moreover, the prediction performance of the same model can be tested on a similar dataset to verify its robustness.

In addition, our study can be further improved by the inclusion of other developing and frontier markets of the world. Moreover, the classical statistical methods have not been applied and compared with the proposed model in this study. Such a comparison can be a valuable research input for the literature on stock markets' prediction. Another significant contribution can be the conduct of an ablation study in which the model's parameters are systematically changed to understand their impact on the overall prediction performance. This can provide a more insightful analysis about the association of the predictive ability of the model with different values of hyperparameters.

### 6.2 Open challenges for stock market prediction

Apart from the model-specific future works delineated above, following are a few open challenges for the research area in stock markets.

#### 6.2.1 Visualization of stock markets' data

The ultimate objective of market forecasts is to decide about the entry and exit points of the stock market and follow trading strategies translating into higher profit. An effective analysis of the equity market is not possible without data visualizations. In this regard, visualization of markets' data particularly at higher frequencies is crucial for all stakeholders, specially technical analysts and day traders. However, effective data visualization with brief but complete depiction of market trends is challenging in technical analysis. Technical analysts frequently use bar charts, candlestick charts, renko charts, etc., for analyzing prices' patterns, discrepancies and trends which often remain unnoticed while going through raw data values. Moreover, highlighting correlations and anomalies among features becomes easier through visual representations for informed and timely decision making. In this regard, another issue is the interpretability of visualizations for a broader audience as technical skills are required to understand, analyze, and make inferences from the given charts and figures. Though most of the market participants have access to the available information, the real challenge is how and when to use that information for translating opportunities into money.

#### 6.2.2 Data mining in stock markets

One important issue for investors is to answer questions of when, where, and how to invest in stock markets' securities. These seem to be simple questions but are complex data-sifting problems in real-time. Effective market forecasts heavily depend upon the applied data mining techniques. Feature extraction, data cleaning and preprocessing, data modeling and evaluation, etc., come under the wider umbrella of data mining. The application of data mining becomes important to filter out potential investment opportunities from a plethora of available market data. With advancements in artificial intelligence and technology, data is accumulated in huge data repositories. The algorithmic trading in developed stock markets generates huge data even at per second frequency. Data mining helps in sifting through big data of the repositories using statistical and mathematical methods, and pattern recognition technologies.

Data integration in data mining is another important area of research for financial analysts. As mentioned earlier, a stock market is a dynamic system affected by a variety of factors in its internal and external environments. In stock markets, data of stock prices and trading volumes come from the market operations. Moreover, there is information gathering from companies' news and performance. The information about domestic and global economies and policies is also to be integrated for market analyses in the short and long run. Information coming from diverse sources is to be formally organized for useful analyses. This is done through data integration that is the process of bringing together data from multiple sources across equity markets to provide accurate, complete, and up-to-date values pivotal for market forecasts. In summary, data mining is basically about connecting dots in large data as per the relevance of the requested information and is a lucrative broad finance research area.

## CRediT authorship contribution statement

**Saima Latif:** Conceptualization, Methodology, Software. **Nadeem Javaid:** Formal analysis, Supervision, Writing – original draft, Writing – review & editing. **Faheem Aslam:** Resources, Software, Supervision. **Abdulaziz Aldegheishem:** Data curation, Funding acquisition, Methodology, Resources. **Nabil Alrajeh:** Data curation, Project administration, Writing – review & editing. **Safdar Hussain Bouk:** Data curation, Software, Validation.

## Declaration of Competing Interest

The authors declare the following financial interests/personal relationships which may be considered as potential competing interests: Abdulaziz Aldegheishem reports financial support was provided by 10.13039/501100002383King Saud University.

## Data Availability

The data used in this study is publicly available on finance.yahoo.com and has been accessed using the YahooFinance API *yfinance* in Python.
